# Editorial: Nuclear medicine in rheumatological diseases' therapy and diagnosis

**DOI:** 10.3389/fmed.2023.1171992

**Published:** 2023-03-14

**Authors:** Giorgio Treglia, Clément Bailly

**Affiliations:** ^1^Division of Nuclear Medicine, Imaging Institute of Southern Switzerland, Ente Ospedaliero Cantonale, Bellinzona, Switzerland; ^2^Faculty of Biomedical Sciences, Università della Svizzera italiana, Lugano, Switzerland; ^3^Faculty of Biology and Medicine, University of Lausanne, Lausanne, Switzerland; ^4^University of Nantes, CHU Nantes, Nantes, France; ^5^Nuclear Medicine Department, University Hospital, Nantes, France

**Keywords:** PET, nuclear medicine, rheumatology, treatment, diagnosis, radionuclides

Rheumatological diseases are common conditions in the general population and their diagnosis in clinical practice is challenging due to their unspecific clinical presentation. Nuclear medicine has a great potential for the management of the rheumatological diseases providing a tool for diagnosis, assessment of prognosis, and treatment efficacy. Furthermore, radiopharmaceuticals may be used as therapeutic options in patients with rheumatological diseases. The application of nuclear medicine techniques in the diagnosis and management of rheumatological diseases relies on the detection of *in vivo* functional abnormalities at an early stage of the diseases, earlier compared to the structural changes detected by conventional imaging (1–7).

This Research Topic comprises 11 articles (four reviews, six original articles, and one study protocol) that highlight the role of nuclear medicine techniques in the management of rheumatological diseases.

The mini-review of Wenger and Schirmer provides an overview on the current use of nuclear medicine imaging modalities in the diagnosis of rheumatological diseases. In particular the authors described that, at present, [^18^F]FDG PET/CT is the hybrid imaging method most often used in rheumatology, in particular for diagnosis of large vessel vasculitis. Despite the current limited value of bone scintigraphy as diagnostic procedure in rheumatological diseases, this nuclear medicine technique has a role before and after radiosynovectomy. In the review of Nassarmadji et al. the evidence on the role of [^18^F]FDG PET/CT for diagnosis, treatment monitoring and follow-up in large vessel vasculitis was summarized. [^18^F]FDG PET/CT is currently not recommended for diagnosis of chronic inflammatory rheumatism including rheumatoid arthritis, spondyloarthritis, and polymyalgia rheumatica. However, this imaging tool seems promising in chronic inflammatory rheumatism as described in the mini-review of De Ponfilly-Sotier et al. as it may provide an overview of systemic involvement occurring in this setting. Lastly, the review of van der Geest et al. illustrates novel PET radiopharmaceuticals that may be useful for diagnosis and treatment monitoring of polymyalgia rheumatica and large vessel vasculitis.

Four original articles included in this Research Topic are focused on polymyalgia rheumatica. French researchers evaluated periarticular [^18^F]FDG uptake scores using PET/CT images to identify polymyalgia rheumatica within a group of patients with rheumatic diseases. The presence of at least three sites with significant uptake identified polymyalgia rheumatica with a sensitivity of 86% and a specificity of 85.5%, suggesting that [^18^F]FDG PET/CT has good performance to identify polymyalgia rheumatica within a population presenting rheumatic diseases (Amat et al.). A research article from the same group demonstrated that machine learning algorithm is useful for diagnosis of polymyalgia rheumatica using [^18^F]FDG PET/CT in a group of patients with inflammatory rheumatisms providing accurate sensitivity and specificity (Flaus et al.).

In a large Belgian retrospective study, researchers scored [^18^F]FDG uptake through visual analysis of PET images taking into account 12 articular regions (scores 0–2 for each articular region). A total skeletal score was calculated by summing the individual scores. These PET scores showed high diagnostic accuracy for the diagnosis of polymyalgia rheumatica (Moreel et al.).

An Italian retrospective study demonstrated that [^18^F]FDG PET/CT performed in patients with polymyalgia rheumatica and persistent increase of acute phase reactants was able to detect persistence of active polymyalgia rheumatica, occult large vessel vasculitis, or other inflammatory diseases (Colaci et al.).

In a retrospective study from China, researchers explored the value of [^18^F]FDG PET/CT for assessing disease activity and for predicting the prognosis of the Adult-onset Still's disease. The authors reported that increased radiopharmaceutical uptake was observed in bone marrow, lymph nodes, and spleen of patients with Adult-onset Still's disease. Taking into account the correlation among PET findings and laboratory inflammatory markers, the authors suggested to use [^18^F]FDG PET/CT for evaluating disease activity and for predicting clinical prognosis of Adult-onset Still's disease (Li et al.).

In another retrospective study, Swiss researchers investigated the emerging role of quantification of ^99m^Tc-labeled diphosphonates uptake by using SPECT/CT in fibrous dysplasia bone lesions; furthermore, the authors correlated SPECT/CT findings with markers of disease activity. They found that bone turnover markers were correlated with diphosphonate uptake on bone scan, suggesting that bone scan, and in particular quantification using SPECT/CT, could be useful to assess the disease burden and to guide treatment and follow-up in patients with fibrous dysplasia (Jreige et al.).

Lastly, this Research Topic also includes a study protocol on a new nuclear medicine imaging method for detecting cartilage disorders. ^99m^Tc-NTP 15-5 is a new radiopharmaceutical that can be used to target proteoglycans, which are a component of the cartilaginous extracellular matrix. Therefore, imaging of proteoglycans could be used for diagnosis, treatment monitoring and follow-up of cartilage disorders. French researchers published a study protocol to assess ^99m^Tc-NTP 15-5 uptake in healthy joints (Thivat et al.).

Findings provided by the articles published in this Research Topic illustrate the current role and future perspectives of nuclear medicine in rheumatological disorders. Currently, among the different nuclear medicine imaging methods, there is a growing evidence supporting the use of [^18^F]FDG PET/CT in several rheumatological diseases even if the role of bone scintigraphy in this setting should not been underestimated (8). Even if [^18^F]FDG uptake is non-specific for inflammatory lesions, however, the distribution and pattern of radiopharmaceutical uptake could help in the differential diagnosis of rheumatological diseases or could demonstrate the true disease extent identifying occult sites on conventional imaging, in addition to identifying sites for biopsy to obtain histological confirmation (8). Furthermore, there is an emerging role of [^18^F]FDG PET/CT to assess the treatment response and to monitor the disease activity (8). Overall, even if there is increasing evidence on the use of [^18^F]FDG PET/CT in the diagnosis of rheumatological diseases, multicentric prospective studies are warranted to fully understand the potential of [^18^F]FDG PET/CT in rheumatological diseases.

About the use of nuclear medicine techniques for therapy of rheumatological diseases, radiosynovectomy involving intra-articular injection of radiolabelled colloids (which induce necrosis and fibrosis of hypertrophic synovial membrane) is a valid, safe and well-tolerated option for treating persistent joint inflammation in rheumatoid arthritis, even if other arthropathies can also benefit from this method. However, radiosynovectomy is still underutilized in rheumatological diseases (9).

## Author contributions

GT and CB drafted the manuscript and revised the final version. Both authors contributed to the article and approved the submitted version.
